# Analytical Performance of the Sysmex HISCL HBsAg Assay and Comparison with the Roche Elecsys HBsAg II Quant Assay in the Quantification of Hepatitis B Surface Antigen

**DOI:** 10.3390/medicina57121307

**Published:** 2021-11-29

**Authors:** Joonhong Park, Taewon Bae, Yonggon Cho, Dalsik Kim, Jaehyeon Lee

**Affiliations:** 1 Department of Laboratory Medicine, Jeonbuk National University Medical School and Hospital, Jeonju 54907, Korea; miziro@jbnu.ac.kr (J.P.); baetw@jbnu.ac.kr (T.B.); choyg@jbnu.ac.kr (Y.C.); dskim@jbnu.ac.kr (D.K.); 2 Research Institute of Clinical Medicine of Jeonbuk National University-Biomedical Research Institute of Jeonbuk National University Hospital, Jeonju 54907, Korea

**Keywords:** comparative evaluation, HISCL HBsAg assay, Elecsys HBsAg II quant assay, hepatitis B surface antigen, HBsAg quantification

## Abstract

*Background and Objectives*: This study aims to estimate the analytical performance of the Sysmex HISCL HBsAg assay and to assess the analytical correlation with the Roche Elecsys HBsAg II quant assay with clinical samples and the WHO International Standard (IS). *Materials and Methods*: The intra-assay precision, linearity, assay limitation, accuracy, and comparative evaluation of the HISCL HBsAg assay were estimated. *Results*: Extrapolating from the plot of the average total allowable error versus the reference value, an accuracy goal of 20% would be achieved around a limit of quantification (LoQ) of 0.014867 IU/mL. The percentage of biases for each level of the WHO IS measured by the two assays were less than 15%, except for the WHO 3rd IS, for which the HISCL HBsAg assay achieved a percentage of bias of 33%. In the comparative evaluation, Passing–Bablok regression analysis did not reveal any significant deviation from linearity between the two assays (y = −48.6998 + 1.9206x; *p* = 0.79 by the CUSUM test for linearity). The mean difference of the quantitative HBsAg level between the two assays was 1762.5 IU/mL in the Bland–Altman plot. *Conclusions*: The HISCL HBsAg assay, with a highly sensitive LoQ of 0.03 IU/mL, showed similar analytical performance in HBsAg quantification to the Elecsys HBsAg II quant assay and may be helpful in obtaining better diagnoses and therapeutic strategies for treating HBV infections.

## 1. Introduction

Infection with the hepatitis B virus (HBV) is a major problem worldwide, with an estimated 248 million chronically infected individuals [1]. The seroprevalence of HBV surface antigen (HBsAg) is 3.61% globally, with a low in the North America and Western Europe (<2%), a moderate in Mediterranean and Eastern Europe (2–8%), a high prevalence in the Africa (>8%) from 0.48% in the Seychelles to 22.38% in the South Sudan [1], and the Western Pacific regions (5.3%) including South Korea, Japan, China, the Philippines, and Vietnam [2]. Identification of HBsAg in plasma or serum has long served as a qualitative diagnostic marker of HBV infection and has proven to be a steady marker that can be used to predict clinical outcomes [3]. HBsAg, a polypeptide of varying size, is a component of the external envelope of the HBV particle and is translated from mRNA with the transcriptional template-active covalently closed circular (ccc) HBV DNA, which is the reflection of the number of infected hepatocytes [4]. Through this relation, the amount of circulating HBsAg is considered to measure indirectly the regulation of infection by the immunological response independent from the antiviral response, which can be assessed by measuring HBV DNA levels in plasma or serum. Plasma or serum HBsAg can be considered to be a surrogate marker of the number of infected cells [3,5]. Monitoring HBsAg levels, in addition to HBV DNA, before and during pegylated interferon or oral nucleoti(si)des therapy can help physicians predict the likely response and implement response-guided therapy algorithms, as recommended in the guidelines, to achieve the optimal outcome for sustained HBsAg loss with or without seroconversion to anti-HBs [6,7]. There is also some evidence suggesting that HBsAg quantification may have value in monitoring the response to nucleoside/nucleotide analog therapy and identifying patients able to achieve a sustained response after terminating treatment, with lower HBsAg levels at the end of treatment being associated with continued remission [6,8,9]. There are two approaches, either definitive suppression of HBV replication with oral nucleoti(si)des or obtained sustained off-therapy virological regulation with a finite course of pegylated interferon. Several studies have reported that baseline HBsAg levels and on treatment HBsAg quantification are candidate predictors of end of sustained virological response and therapeutic response [10,11,12]. Thus, the accuracy and quality of HBsAg quantification should be important considerations in test selection for HBV patients. Among the commercially available HBsAg quantitative assays, the Sysmex HISCL HBsAg assay (Sysmex Corporation, Kobe, Japan) can not only determine the presence of HBsAg but also quantitatively measure the amount of HBsAg with the widest available dynamic range (from 0.03 to 2500 IU/mL) without dilution.

This study aims to estimate the analytical performance of the Sysmex HISCL HBsAg assay and to compare it to that of the Roche Elecsys HBsAg II quant assay (Roche Diagnostics, Mannheim, Germany) via analytical correlation with clinical samples and the WHO International Standard (IS).

## 2. Materials and Methods

### 2.1. Samples

The study was approved by the Institutional Review Board of Jeonbuk National University Hospital (IRB No. 2014-10-025). Informed consent was waived because patient information was not required to evaluate in vitro diagnostic test. A total of 437 serum samples were collected from HBV-infected patients, those who had been undergoing follow-up observations for hepatitis and cirrhosis at the Jeonbuk National University Hospital (Jeonju, Korea) between September 2014 and August 2015. Samples were selected based on the results from an ADVIA Centaur HBsAgII assay with ADVIA Centaur XP (Siemens Diagnostics, Tarrytown, NY, USA). Serum samples left over after the testing were aliquoted into 8 to 12 microtubes and stored immediately at −20 °C until testing. The WHO 3rd IS for HBsAg (HBV genotype B4, HBsAg subtypes ayw1/adw2; NIBSC code 12/226) and the WHO 1st international reference panel (IRP) for HBsAg (HBV genotype A, HBsAg subtype adw2; NIBSC code 03/262) (IU/vial: 8.25; 2.06; 0.52; 0.13; 0.00) (Hertfordshire, United Kingdom) were used.

### 2.2. Hepatitis B Virus Surface Antigen Quantification

The HISCL HBsAg assay measures HBsAg based on the chemiluminescence enzyme immunoassay (CLEIA) method with CDP-Star^®^ chemiluminescent substrate and has the following characteristics. Briefly, biotinylated anti-HBs monoclonal antibodies (mouse) specifically react with HBsAgs in the sample and bind to streptavidin-coated magnetic particles (MPs). After bound/free (B/F) separation, alkaline phosphatase (ALP)-labeled anti-HBs monoclonal antibodies (mouse) specifically bind to the HBs antigen on an MP. Then, the ALP on the MP breaks down the CDP-Star^®^ substrate to an excited intermediate, which produces a luminescent signal. Assay samples were tested undiluted, with an analytical measurement range of 0.03–2500 IU/mL without predilution. On the other hand, the Elecsys HBsAg II quant assay is a two-step sandwich electrochemiluminescence immunoassay (ECLIA) with improved sensitivity for the in vitro quantitative determination of HBsAg. All samples were tested using Cobas e601/e602 at a default 1:400 dilution with the serum diluent provided by the manufacturer. A mixture of Biotinylated monoclonal (mouse) and polyclonal (sheep) antibodies was used as capture antibodies. Sequentially, monoclonal (mouse) and polyclonal (sheep) anti-HBsAg antibodies labeled with Tris(2,2′-bipyridyl)ruthenium(II)-complex were used as conjugate antibodies. If the result revealed a cutoff index (COI) of >1, then the final result was COI × 400. If COI < 1, then the sample was retested undiluted, and the final result was that of the retest. If COI ≥ 1000, then the sample was retested at a 1:8000 dilution, and the final result was COI × 8000.

### 2.3. Performance Evaluation of HISCL HBsAg Assay

To assess the intra-assay precision according to CLSI EP15-A, one set of three HISCL HBsAg and Elecsys HBsAg II quant assay controls, each consisting of low, medium, and high positive controls, was tested in duplicate, once a day for 20 consecutive days. In addition, pooled sera were adjusted to three different levels, low, medium, and high, and tested in duplicate, twice a day for 20 consecutive days. Coefficients of variation (CVs, %) were calculated to estimate the intra-assay precision above the lower limit of the measuring range according to the manufacturer’s instructions.

To evaluate the linearity according to CLSI EP06-A in which 7 to 11 or more points specific concentrations across the anticipated measuring range should be prepared when establishing linear ranges for new methods, pooled sera were adjusted from the upper limit of 2220.39 IU/mL to near the lower limit of the measuring range, 0.03 IU/mL. Briefly, dilution series were made with 15 two-fold dilution steps. Thus, 16 serially diluted pooled serum samples ranging from 2220.39 to 0.07 (×1/32,768) IU/mL were measured in 5 replicates of each level. 

To verify the assay limitations of the HISCL HBsAg assay, the limit of blank (LoB), limit of detection (LOD), and limit of quantification (LoQ) were validated according to CLSI EP17-A2. Two reagent lots, ZS4101 and ZS4107, were used. A total of 60 replicates of blank was measured to verify the LoB of this assay in 20 replicates per five blanks, one a day for 3 consecutive days. To verify the LoD and LoQ of this assay, five serial dilution series from the WHO 3rd IS for HBsAg were tested: 0.015, 0.012, 0.009, 0.006, and 0.003 IU/mL in 20 replicates per five samples, once a day for 3 consecutive days. The lowest concentration at which the CV and the bias were less than or equal to the total allowable error (TE) was considered the LoQ. The LoQ accuracy goal was a TE of 20%.

To compare the analytical accuracy between the two assays, the WHO 3rd IS (47.3 IU/mL) and WHO 1st IRP (dilutional panels of 8.25, 2.06, 0.52, 0.13, and 0 IU/mL) for HBsAg were tested in duplicate a day for 5 consecutive days.

### 2.4. Comparison between the HISCL HBsAg and Elecsys HBsAg II Quant Assays

To assess the quantitative correlation between the two assays, 63 serum samples with a signal-to-cutoff ratio > 50 as an index for not performing confirmatory testing were sequentially chosen at random and stored at −20 °C for up to three days before reanalysis. Ten to 15 different samples were retested by the two assays, once a day for 5 consecutive days.

For qualitative estimation near the cutoff level between the two assays, 374 serum samples with signal-to-cutoff ratios ranging from 1 to 50 as an index required for confirmatory testing were sequentially tested. In particular, the presence of HBsAg was confirmed with an ADVIA Centaur HBsAg confirmatory assay, additional HBV marker assays, or HBV DNA quantification determined by a COBAS AmpliPrep/COBAS TaqMan HBV assay (Roche Molecular Diagnostics, Pleasanton, CA, USA). Fifteen to 20 different samples were retested by the HISCL HBsAg assay, once a day for 20 consecutive days.

### 2.5. Statistical Analysis

Descriptive statistics are shown as the means ± standard deviations (SD) or as medians and interquartile ranges, as appropriate. The inter-rater agreement between the two assays was calculated and interpreted according to the guidelines of Cohen’s kappa coefficient as follows: values ≤ 0 as indicating no agreement and 0.01–0.20 as none to slight, 0.21–0.40 as fair, 0.41–0.60 as moderate, 0.61–0.80 as substantial, and 0.81–1.00 as almost perfect agreement. The relationship between quantitative variables was assessed by means of Passing–Bablok regression analysis and the Bland–Altman plot method. Statistical analyses were performed using MedCalc version 17.6 (MedCalc, Ostend, Belgium) and the diagnostic test evaluation calculator provided by MedCalc (https://www.medcalc.org/calc/diagnostic_test.php; accessed on 14 March 2021). *p* values of <0.05 were considered statistically significant.

## 3. Results

### 3.1. Performance Evaluation of the HISCL HBsAg Assay

Performance characteristic information for the two HBsAg quantitative assays provided by the manufacturers is summarized in Table 1.

For the low, medium, and high levels of quality controls and the pooled sera assessed by the HISCL HBsAg assay, the CV of each result was less than the 15% reproducibility provided by the manufacturer. The precision results are shown in Table 2.

**Figure 1:**
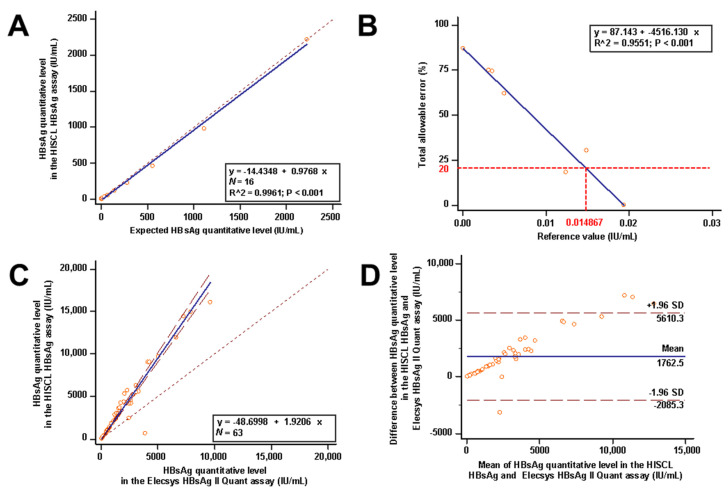
Analytical performance evaluation of the HISCL HBsAg assay (**A**,**B**) and comparative evaluation between the HISCL HBsAg and Elecsys HBsAg II quant assays (**C**,**D**). (**A**) Linearity analysis of the HISCL HBsAg assay using serially diluted samples ranged from 2220.39 to 0.07 IU/mL. The best fit regression equation for the HISCL HBsAg assay was y = −14.4348 + 0.9768x. The dotted line represents the equality line. Abbreviation: HBsAg, hepatitis B surface antigen. (**B**) Limit of quantification of the HISCL HBsAg assay determined using five diluted samples: 0.015, 0.012, 0.009, 0.006, and 0.003 IU/mL. Extrapolating from the plot of the average TE versus the reference value, the accuracy goal of 20% would be achieved around a limit of quantification of 0.014867 IU/mL. Abbreviation: HBsAg, hepatitis B surface antigen. (**C**) Passing–Bablok regression analysis between the HISCL HBsAg and Elecsys HBsAg II quant assays. Passing–Bablok regression analysis did not reveal any significant deviation from linearity between the two assays (y = −48.6998 + 1.9206x; *p* = 0.79 by the CUSUM test for linearity). The dashed lines represent the 95% confidence interval, and the dotted line represents the equality line. Abbreviation: HBsAg, hepatitis B surface antigen. (**D**) Bland–Altman plot analysis between the HISCL HBsAg and Elecsys HBsAg II quant assays. The mean difference of the quantitative HBsAg level between the two assays was 1762.5 IU/mL in the Bland–Altman plot. The straight dashed lines represent the mean difference ± 1.96 SD. Abbreviation: HBsAg, hepatitis B surface antigen.

In an outlier analysis of the dataset consisting of HBsAg quantitative testing, no outliers were observed; therefore, all the data were accepted. HBsAg levels measured by the HISCL HBsAg assay correlated with the calculated HBsAg interval ranging from 0.07 to 2220.39 IU/mL for HBsAg quantification. A linear coefficient was found in regression analysis (R^2^ = 0.9961; *p* < 0.001). The best fit regression equation for the HISCL HBsAg assay was y = −14.4348 + 0.9768x. The result of the linearity assessment is shown in Figure 1A.

The LoD of the HISCL HBsAg assay was calculated as 0.010342 IU/mL from LoD = LoB + cpSDL (cp =1.658824). Visual inspection of these results shows that none of the low-level samples except the expected value of 0.012 yielded a TE meeting the accuracy goal of 20%. Extrapolating from the plot of the average TE versus the reference value, the accuracy goal of 20% would be achieved around a limit of quantification of 0.014867 IU/mL (Figure 1B).

The CV ranges of the HISCL HBsAg assay and Elecsys HBsAg II quant assay were 1.35 to 4.30% and 3.66 to 5.20%, respectively. The percentage of biases for each level of the WHO IS measured by the two assays were less than 15%, except for the WHO 3rd IS measured by the HISCL HBsAg assay (33%). The results of the accuracy assessment are shown in Table 3.

### 3.2. Comparison between the HISCL HBsAg and Elecsys HBsAg II Quant Assays

The results were highly correlated, and Passing–Bablok regression analysis did not reveal any significant deviation from linearity between the two assays (r = 0.918, 95% CI: 0.861 to 0.952, *p* < 0.0001 by Spearman’s rank correlation; linear regression equation, y = −48.6998 + 1.9206x; *p* = 0.79 by the CUSUM test for linearity) (Figure 1C). The mean difference of the quantitative HBsAg level between the two assays was 1762.5 IU/mL (95% CI: −3025.4 to 1215.6) and proportional error was observed between the two immunoassays in the Bland–Altman plot (Figure 1D). The delta percentage difference between the two assays was −10 to 159%.

The Kappa agreement between the two assays was almost perfect, with nine discrepancies (HISCL (+) Elecsys (−), 7; HISCL (−) Elecsys (+), 2). The concordance rates of the HISCL HBsAg and Elecsys HBsAg II quant assays relative to the confirmed results were 98.13% and 96.79%, respectively. When the HBsAg seroprevalence of 5.3% from the Western Pacific region, including South Korea, was applied, the HISCL HBsAg assay was superior to the Elecsys HBsAg II quant assay in terms of predictive value and accuracy. The qualitative estimation results are summarized in Table 4.

## 4. Discussion

In this study, the HISCL HBsAg assay developed assay based on the CLEIA principle was compared to the Elecsys HBsAg II quant assay, a widely used assay based on the ECLIA principle. Because of the excellent analytical performance reported in previous studies [13,14,15,16,17,18], the Elecsys HBsAg II was selected as a comparative assay in this study. The HISCL HBsAg assay has excellent analytical performance, with an estimated LoQ lower than that claimed by the manufacturer when TE was defined as the accuracy goal of 20%. In a recent study, these two assays had good performance in screening four common blood-borne pathogens, including HBV, and were found to be comparable and considered adequate for clinical use [18]. Other studies have also indicated that HISCL HBsAg assay is comparable and considered adequate for clinical use in the highly sensitive detection of HBsAg [19,20,21]. However, in our study, the CLEIA-based assay showed better bias than the Elecsys HBsAg II quant assay when the accuracy was verified using WHO 1st IRPs ranging from 0.13 to 8.25 IU/mL and blank but not at the assayed level of 47.3 IU/mL (Table 3). HBsAg quantification was highly correlated between the HISCL HBsAg and Elecsys HBsAg II quant assays; however, the quantification was estimated to be higher for the former assay depending on the HBsAg concentration. A similar phenomenon was observed when accuracy verification was performed using the WHO IS. The qualitative concordance of the HISCL HBsAg assay (98.13%) was better than that of the Elecsys HBsAg II quant assay (96.79%) in confirmed samples near the cutoff level. Indeed, for five chronic HBV patients, 0.03, 0.04, and 0.06 IU/mL were measured for one, two, and two patients, respectively by the HISCL HBsAg assay; however, all measurements were < 0.05 IU/mL with the Elecsys HBsAg II quant assay, leading to false negatives. This was observed for all very low-level samples; the assay limitation for very low viremic levels can lead to such qualitative discrepancies.

Our main concern is that a difference in correlation between two immunoassays was observed in a concentration-dependent manner. The differences in the results from the comparative studies might be due to diluent matrix effects or interactions between the components of blood collection tubes and blood samples [22]. Concentrating samples with HBsAg concentrations beyond the maximum limit of detection might be highly subjective because of dilution effects. In the present study, the sample dilution did not seem to influence the data because autodilution is not applied initially for the HISCL HBsAg assay; thus, the matrix effect of the diluent for this assay was minimal. Second, these quantitative deviations and qualitative disagreements may be explained by the differences in the detection mechanism between CLEIA for HISCL HBsAg assay and ECLIA for Elecsys HBsAg II assay, depending on the method of generating chemiluminescence [23]. While HISCL HBsAg assay uses chemical reactions to generate chemiluminescence following antibody-antigen binding, Elecsys HBsAg II assay uses electrochemical reactions to generate chemiluminescence. However, both techniques are rapid and specific [18]. When comparative evaluation of the four method principles for HBsAg level, there was no significant discrepancy between HISCL HBsAg and Elecsys HBsAg II assays and they were suitable for the quantitative determination of HBsAg [16]. When comparing between two widely used quantitative HBsAg assays, Elecsys HBsAg II and Architect HBsAg assays, the quantitative Elecsys HBsAg II assay reliably determined serum HBsAg levels in a wide range of samples and showed very high correlation with the Architect HBsAg assay [13]. Moreover, the results of the quantitative Elecsys HBsAg II were highly correlated with those of the Architect HBsAg assay in patients carrying both HBsAg and anti-HBs antibodies, but differences were observed between the platforms in samples with low HBsAg levels [17]. Similarly, the Elecsys HBsAg II assay is wholly capable of quantifying serum HBsAg levels with very high correlation and precision, compared to those of the Architect HBsAg QT assay in Human Immunodeficiency Virus (HIV)-HBV-coinfected patients [14]. ECLIA yields a specific chemiluminescence reaction initiated by electrochemistry on the surface of the electrode, which is easier to control and more accurate than when initiated by simple mixing of the compound, as with CLEIA [24]. Third, the composition of the antibody was different between two immunoassays. Unlike HISCL HBsAg assay using only anti-HBs monoclonal mouse antibodies, Elecsys HBsAg II assay uses a mixture of Biotinylated monoclonal (mouse) and polyclonal (sheep) (Table 1). Polyclonal antibodies are employed to increase the sensitivity supposedly in the commercial kits as exampled above. However, using polyclonal antibodies causes problems of inconsistency of production and quality [25]. Therefore, the variability in HBsAg measurements might be mainly due to harmonization problems.

Other limitation of our study is that the influence of the different HBV genotypes and HBsAg escape mutations on HBsAg quantification was not estimated. In Korea, prevailing HBV genotypes are genotype C2 or a mixed pattern of genotypes B and C, while other genotypes rarely occur [26]. Therefore our performance data was not applicable for quantitating HBsAg with different genotypes. The HBsAg levels obtained by different immunoassays now need comparing and the relationships between levels of HBsAg and HBV DNA alongside HBsAg and genotype should be evaluated. Thus, the comparative evaluation between two assays should be performed with many samples consist of multiple genotypes because the genotype-dependent correlation has already been reported in the HBsAg quantification [27,28,29]. By contrast with previous studies, HBsAg quantitation is not affected by HBV genotype, the observed association between levels of HBsAg and HBV DNA seems genotype dependent [15]. Although HBV genotype C prevails predominantly irrespective of their clinical stages of liver disease and geographic origin among chronic carriers of the virus in Korea such discrepancy between two assays could be partly explained by different clinical situation in the population of patients that we did not take into account. In addition, several studies reported HBV reactivation cases in patients positive for anti-HBs antibodies contribute to the hypothesis that immune-escape HBsAg mutations confer risk of HBV reactivation [30,31,32]. Further studies should be required to better characterize the association of serum HBsAg qualitative levels with the various clinical settings of HBV infection with different HBV genotypes and HBsAg escape mutations.

Finally, analytical performance evaluation of HISCL HBsAg assay was performed only in 36 samples with an HBsAg level between 1000 and 10,000 IU/mL and seven samples with an HBsAg level > 10,000 IU/mL. Although HISCL HBsAg assay can measure a relatively wide range up to 2500 IU/mL without sample dilution, high active HBV patients often show HBsAg titers over upper measuring limit of 2500 IU/mL. For similar reason, Intra-assay precision of HISCL HBsAg assay at the concentration exceeding 2500 IU/mL should be evaluated. Because monitoring of serum HBsAg levels may be useful for predicting virological response or HBeAg loss/seroconversion in HBeAg-positive patients, but not in HBeAg-negative patients [33], additional comparative investigation should be required using high HBsAg level from 10,000 to 100,000 IU/mL.

To resolve these limitations, using the reference material for evaluating commercial kits would be very helpful for harmonization or standardization of the assay, and we believe it is needed for accurate diagnoses and follow-ups of treatment for HBV infection [34]. Ideally one would also analyze longitudinal samples from some patients during antiviral therapy with effect on HBsAg such as pegylated interferon or oral nucleoti(si)des therapy.

## 5. Conclusions

The HISCL HBsAg assay, with a highly sensitive LoQ of 0.03 IU/mL, showed similar analyti-cal performance in HBsAg quantification to the Elecsys HBsAg II quant assay and may be helpful to better diagnoses and therapeutic strategies in treating HBV infections. With random access capability and full automation, the HISCL HBsAg assay is sensitive and specific, accurately quantifies HBsAg levels in HBV infection and is suitable for high-throughput, quantitative HBsAg monitoring in large hospital laboratories.

## Figures and Tables

**Table 1:** Summary of performance characteristic information of two quantitative HBsAg assays provided by the manufacturer.

Characteristics	HISCL HBsAg Assay	Elecsys HBsAg II Quant Assay
Manufacturer	Sysmex Corporation	Roche Diagnostics
Principle of operation	CLEIA	ECLIA
Unit	IU/mL (quantitative)	IU/mL (quantitative)
Capture antibodies	Biotinylated monoclonal antibodies (mouse)	A mixture of Biotinylated monoclonal (mouse) and polyclonal (sheep) antibodies
Conjugate antibodies	Alkaline phosphatase monoclonal antibodies (mouse)	Monoclonal (mouse) and polyclonal (sheep) anti-HBsAg antibodies labeled with Tris(2,2′-bipyridyl)ruthenium(II)-complex
Duration of assay (minutes)	17	18
Sample volume (μL)	20	50
Limit of quantitation (IU/mL)	≥0.03	≥0.05
Reproducibility	CV of each positive control < 15%	CV < 3.2% in Cobas e 601/e602
Analytical measuring range (theoretical)	0.03–2500 IU/mL (auto dilution available)	0.05–130 IU/mL (pre-dilution applied)
Traceability of values assigned to calibrators	Second WHO IS, NIBSC 00/588	Second WHO IS, NIBSC 00/588

CLEIA, chemiluminescent enzyme immunoassay; ECLIA, electrochemiluminescence immunoassay. 5–13,000 IU/mL for 100-fold diluted samples in Elecsys 2010 and Cobas e411 analyzers. 20–52,000 IU/mL for 400-fold diluted samples in Cobas e601, Cobas e602, and MODULAR ANALYTICS E170 analyzers.

**Table 2:** Intra-assay precisions of the HISCL HBsAg and Elecsys HBsAg II Quant assays.

Materials	HISCL HBsAg Assay		Elecsys HBsAg II Quant Assay	
	Mean, SD (IU/mL)	CV (%)	Mean, SD (IU/mL)	CV (%)
Controls				
Low *	<0.03, NA	NA	<0.05, NA	NA
Medium	635.97, 62.75	9.87	419.09, 23.05	5.50
High	1355.93, 151.28	11.16	657.03, 35.81	5.45
Pooled sera				
Level 1 *	<0.03, Not available	Not available	Not done	Not done
Level 2	668.47, 13.90	2.08	Not done	Not done
Level 3	1474.27, 47.55	3.23	Not done	Not done

NA, not available. * Value below limit of quantitation was described as “< limit of quantitation” provided by manufacturer.

**Table 3:** Accuracy verification of the HISCL HBsAg and Elecsys HBsAg II Quant assays using WHO international standard.

Standard Materials	Assayed Level (IU/mL)	HISCL HBsAg Assay			Elecsys HBsAg II Quant Assay		
		Mean, SD (IU/mL)	CV (%)	Bias (IU/mL)	Mean, SD (IU/mL)	CV (%)	Bias (IU/mL)
WHO 3rd IS *	47.3	63.10, 0.85	1.35	15.8 (33%)	43.51, 2.21	5.08	−3.79 (−8%)
WHO 1st IS ^†^							
A	8.25	8.48, 0.20	2.41	0.23 (3%)	7.33, 0.28	3.83	−0.92 (−11%)
B	2.06	1.97, 0.05	2.32	−0.09 (−4%)	1.91, 0.07	3.66	−0.15 (−7%)
C	0.52	0.46, 0.01	1.90	−0.06 (−12%)	0.46, 0.02	4.47	−0.06 (−12%)
D	0.13	0.11, 0.00	4.30	−0.02 (−15%)	0.11, 0.01	5.2	−0.02 (−15%)
E ^‡^	0.00	<0.03, NA	NA	NA	<0.05, NA	NA	NA

NA, not available. * WHO 3rd international standard for HBsAg (HBV genotype B4, HBsAg subtypes ayw1/adw2; NIBSC code 12/226). ^†^ WHO 1st international reference panel (IRP) for HBsAg (HBV genotype A, HBsAg subtype adw2; NIBSC code 03/262) (IU/vial: 8.25; 2.06; 0.52; 0.13) (Hertfordshire, United Kingdom). ^‡^ Value below limit of quantitation was described as “< limit of quantitation” provided by manufacturer.

**Table 4:** Comparison of qualitative estimation between two assays using 374 samples ranging from 1 to 50 Index required for the ADVIA Centaur HBsAg Confirmatory assay.

Statistics	HISCL HBsAg Assay	Elecsys HBsAg II Quant Assay
Kappa agreement	Almost perfect (Kappa, 0.848; 95% CI, 0.752 to 0.945)	
Concordance rate (%)	98.13	96.79
True positive (*N*)	33	28
True negative (*N*)	334	334
False positive (*N*)	2	2
False negative (*N*)	5	10
Sensitivity (%)	86.84 (95% CI, 71.91 to 95.59)	73.68 (95% CI, 56.90 to 86.60)
Specificity (%)	99.40 (95% CI, 97.87 to 99.93)	99.40 (95% CI, 97.87 to 99.93)
Positive likelihood ratio	145.89 (95% CI, 36.44 to 584.18)	123.79 (95% CI, 30.69 to 499.39)
Negative likelihood ratio	0.13 (95% CI, 0.06 to 0.30)	0.26 (95% CI, 0.16 to 0.45)
Positive predictive value * (%)	89.01 (95% CI, 66.92 to 97.01)	87.3 (95% CI, 63.01 to 96.52)
Negative predictive value * (%)	99.27 (95% CI, 98.36 to 99.68)	98.55 (95% CI, 97.56 to 99.14)
Accuracy * (%)	98.74 (95% CI, 97.02 to 99.61)	98.05 (95% CI, 96.08 to 99.20)

* HBV surface antigen (HBsAg) seroprevalence of 5.26% in the Western Pacific region including South Korea, Japan, China, Philippines, and Vietnam.

## Data Availability

The data presented in this study are available on request from the corresponding author.

## References

[1] Schweitzer A., Horn J., Mikolajczyk R.T., Krause G., Ott J.J. (2015). Estimations of worldwide prevalence of chronic hepatitis B virus infection: A systematic review of data published between 1965 and 2013. Lancet.

[2] Sarin S.K., Kumar M., Lau G.K., Abbas Z., Chan H.L., Chen C.J., Chen D.S., Chen H.L., Chen P.J., Chien R.N. (2016). Asian-Pacific clinical practice guidelines on the management of hepatitis B: A 2015 update. Hepatol. Int..

[3] Martinot-Peignoux M., Lapalus M., Asselah T., Marcellin P. (2014). HBsAg quantification: Useful for monitoring natural history and treatment outcome. Liver Int..

[4] Lee J.M., Ahn S.H. (2011). Quantification of HBsAg: Basic virology for clinical practice. World J. Gastroenterol..

[5] Ganem D., Prince A.M. (2004). Hepatitis B virus infection--natural history and clinical consequences. N. Engl. J. Med..

[6] Martinot-Peignoux M., Asselah T., Marcellin P. (2013). HBsAg quantification to predict natural history and treatment outcome in chronic hepatitis B patients. Clin. Liver Dis..

[7] Chan H.L., Wong V.W., Chim A.M., Chan H.Y., Wong G.L., Sung J.J. (2010). Serum HBsAg quantification to predict response to peginterferon therapy of e antigen positive chronic hepatitis B. Aliment. Pharmacol. Ther..

[8] Wang C.C., Tseng T.C., Wang P.C., Lin H.H., Kao J.H. (2014). Baseline hepatitis B surface antigen quantitation can predict virologic response in entecavir-treated chronic hepatitis B patients. J. Formos. Med. Assoc..

[9] Peng C.Y., Lai H.C., Su W.P., Lin C.H., Chuang P.H., Chen S.H., Chen C.H. (2017). Early hepatitis B surface antigen decline predicts treatment response to entecavir in patients with chronic hepatitis B. Sci. Rep..

[10] Sonneveld M.J., Rijckborst V., Cakaloglu Y., Simon K., Heathcote E.J., Tabak F., Mach T., Boucher C.A., Hansen B.E., Zeuzem S. (2012). Durable hepatitis B surface antigen decline in hepatitis B e antigen-positive chronic hepatitis B patients treated with pegylated interferon-α2b: Relation to response and HBV genotype. Antivir. Ther..

[11] Takkenberg R.B., Jansen L., de Niet A., Zaaijer H.L., Weegink C.J., Terpstra V., Dijkgraaf M.G., Molenkamp R., Jansen P.L., Koot M. (2013). Baseline hepatitis B surface antigen (HBsAg) as predictor of sustained HBsAg loss in chronic hepatitis B patients treated with pegylated interferon-α2a and adefovir. Antivir. Ther..

[12] Wu S., Luo W., Wu Y., Chen H., Peng J. (2020). HBsAg quantification predicts off-treatment response to interferon in chronic hepatitis B patients: A retrospective study of 250 cases. BMC Gastroenterol..

[13] Wursthorn K., Jaroszewicz J., Zacher B.J., Darnedde M., Raupach R., Mederacke I., Cornberg M., Manns M.P., Wedemeyer H. (2011). Correlation between the Elecsys HBsAg II assay and the Architect assay for the quantification of hepatitis B surface antigen (HBsAg) in the serum. J. Clin. Virol..

[14] Maylin S., Boyd A., Delaugerre C., Zoulim F., Lavocat F., Simon F., Girard P.M., Lacombe K. (2012). Comparison between Elecsys HBsAg II and architect HBsAg QT assays for quantification of hepatitis B surface antigen among patients coinfected with HIV and hepatitis B virus. Clin. Vaccine Immunol..

[15] Tuaillon E., Mondain A.M., Nagot N., Ottomani L., Kania D., Nogue E., Rubbo P.A., Pageaux G.P., Van de Perre P., Ducos J. (2012). Comparison of serum HBsAg quantitation by four immunoassays, and relationships of HBsAg level with HBV replication and HBV genotypes. PLoS ONE.

[16] Liu C., Chen T., Lin J., Chen H., Chen J., Lin S., Yang B., Shang H., Ou Q. (2014). Evaluation of the performance of four methods for detection of hepatitis B surface antigen and their application for testing 116,455 specimens. J. Virol. Methods.

[17] Liu W., Hu Y., Yang Y., Hu T., Wang X. (2015). Comparison of two immunoassays for quantification of hepatitis B surface antigen in Chinese patients with concomitant hepatitis B surface antigen and hepatitis B surface antibodies. Arch. Virol..

[18] Xu W., Tong Y., Li Y. (2019). Comparison of Roche Elecsys and Sysmex HISCL immunoassays for the screening of common blood-borne pathogens. Ann. Transl. Med..

[19] Feng S., Wei B., Rao C., Wang T., Xiao Y., Tao C., Wang L. (2016). Clinical Evaluation of the Newly Developed HISCL-5000 Analyzer on Detection of Hepatitis B Virus Markers in West China Hospital. Clin. Lab..

[20] Deguchi M., Kagita M., Yoshioka N., Tsukamoto H., Takao M., Tahara K., Maeda I., Hidaka Y., Yamauchi S., Kaneko A. (2018). Evaluation of the highly sensitive chemiluminescent enzyme immunoassay “Lumipulse HBsAg-HQ” for hepatitis B virus screening. J. Clin. Lab. Anal..

[21] Gencay M., Seffner A., Pabinger S., Gautier J., Gohl P., Weizenegger M., Neofytos D., Batrla R., Woeste A., Kim H.S. (2018). Detection of in vivo hepatitis B virus surface antigen mutations-A comparison of four routine screening assays. J. Viral. Hepat..

[22] Bowen R.A., Remaley A.T. (2014). Interferences from blood collection tube components on clinical chemistry assays. Biochem. Med..

[23] Cinquanta L., Fontana D.E., Bizzaro N. (2017). Chemiluminescent immunoassay technology: What does it change in autoantibody detection?. Auto. Immun. Highlights.

[24] Blackburn G.F., Shah H.P., Kenten J.H., Leland J., Kamin R.A., Link J., Peterman J., Powell M.J., Shah A., Talley D.B. (1991). Electrochemiluminescence detection for development of immunoassays and DNA probe assays for clinical diagnostics. Clin. Chem..

[25] Kim S.H. (2017). ELISA for Quantitative Determination of Hepatitis B Virus Surface Antigen. Immune Netw..

[26] Bae S.H., Yoon S.K., Jang J.W., Kim C.W., Nam S.W., Choi J.Y., Kim B.S., Park Y.M., Suzuki S., Sugauchi F. (2005). Hepatitis B virus genotype C prevails among chronic carriers of the virus in Korea. J. Korean Med. Sci..

[27] Sugiyama M., Tanaka Y., Kato T., Orito E., Ito K., Acharya S.K., Gish R.G., Kramvis A., Shimada T., Izumi N. (2006). Influence of hepatitis B virus genotypes on the intra- and extracellular expression of viral DNA and antigens. Hepatology.

[28] Brunetto M.R., Moriconi F., Bonino F., Lau G.K., Farci P., Yurdaydin C., Piratvisuth T., Luo K., Wang Y., Hadziyannis S. (2009). Hepatitis B virus surface antigen levels: A guide to sustained response to peginterferon alfa-2a in HBeAg-negative chronic hepatitis B. Hepatology.

[29] Wursthorn K., Jung M., Riva A., Goodman Z.D., Lopez P., Bao W., Manns M.P., Wedemeyer H., Naoumov N.V. (2010). Kinetics of hepatitis B surface antigen decline during 3 years of telbivudine treatment in hepatitis B e antigen-positive patients. Hepatology.

[30] Colson P., Borentain P., Coso D., Motte A., Aurran-Schleinitz T., Charbonnier A., Stoppa A.M., Chabannon C., Serrero M., Bertrand J. (2015). Hepatitis B virus reactivation in HBsAg-negative patients is associated with emergence of viral strains with mutated HBsAg and reverse transcriptase. Virology.

[31] Salpini R., Colagrossi L., Bellocchi M.C., Surdo M., Becker C., Alteri C., Aragri M., Ricciardi A., Armenia D., Pollicita M. (2015). Hepatitis B surface antigen genetic elements critical for immune escape correlate with hepatitis B virus reactivation upon immunosuppression. Hepatology.

[32] Inoue J., Kondo Y., Wakui Y., Kogure T., Morosawa T., Fujisaka Y., Umetsu T., Takai S., Nakamura T., Shimosegawa T. (2016). Reactivation of resolved hepatitis B virus infection with immune escape mutations after long-term corticosteroid therapy. Clin. J. Gastroenterol..

[33] Chen C.H., Chiu Y.C., Lu S.N., Lee C.M., Wang J.H., Hu T.H., Hung C.H. (2014). Serum hepatitis B surface antigen levels predict treatment response to nucleos(t)ide analogues. World J. Gastroenterol..

[34] Wilkinson D.E., Seiz P.L., Schüttler C.G., Gerlich W.H., Glebe D., Scheiblauer H., Nick S., Chudy M., Dougall T., Stone L. (2016). International collaborative study on the 3rd WHO International Standard for hepatitis B surface antigen. J. Clin. Virol..

